# Supplementary material for: Copy number normalization distinguishes differential signals driven by copy number differences in ATAC-seq and ChIP-seq
Source: BMC Genomics. 2025 Mar 28;26:306. doi: 10.1186/s12864-025-11442-y (PMC11951689; doi:10.1186/s12864-025-11442-y)
Supplement: Supplementary file 1 — Additional file 1. Copy number ratio in Bloom syndrome cell line relative to wild type cell line for each chromosome. [file 12864_2025_11442_MOESM1_ESM.pdf]

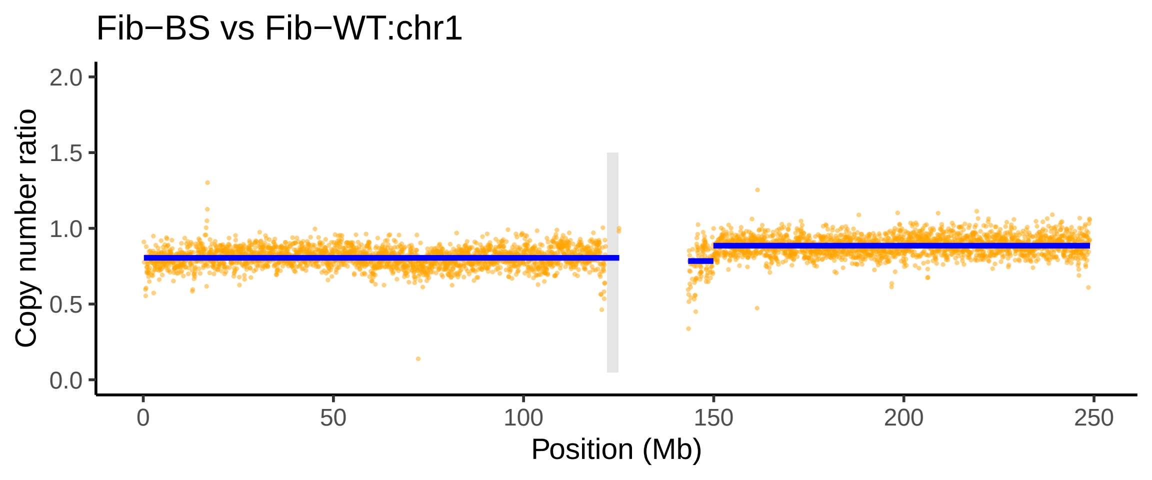

Fib−BS vs Fib−WT:chr1
Copy number ratio
2.0
1.5
1.0
0.5
0.0
0
50
100
150
200
250
Position (Mb)

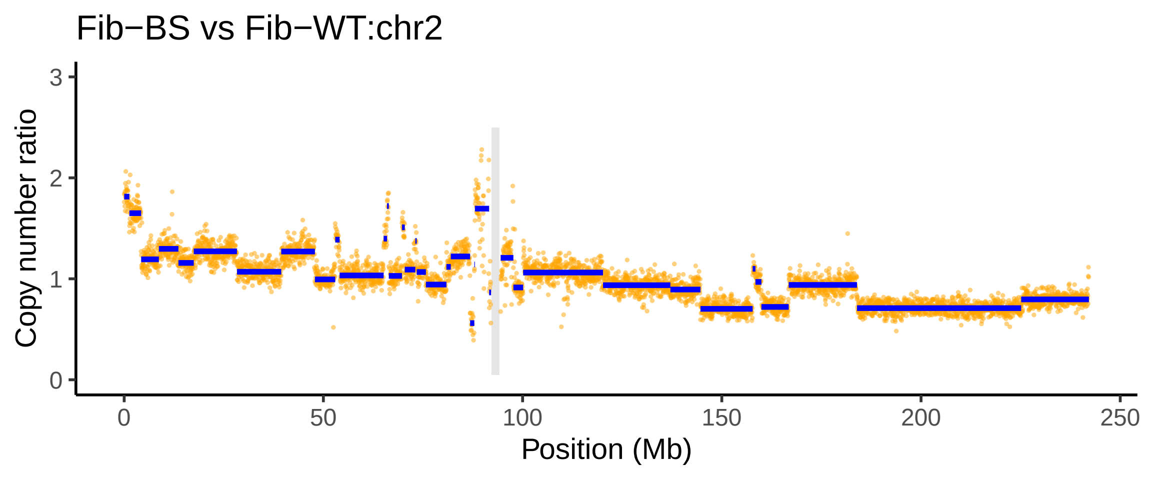
Fib−BS vs Fib−WT:chr2
Copy number ratio
0
1
2
3
0
50
100
150
200
250
Position (Mb)

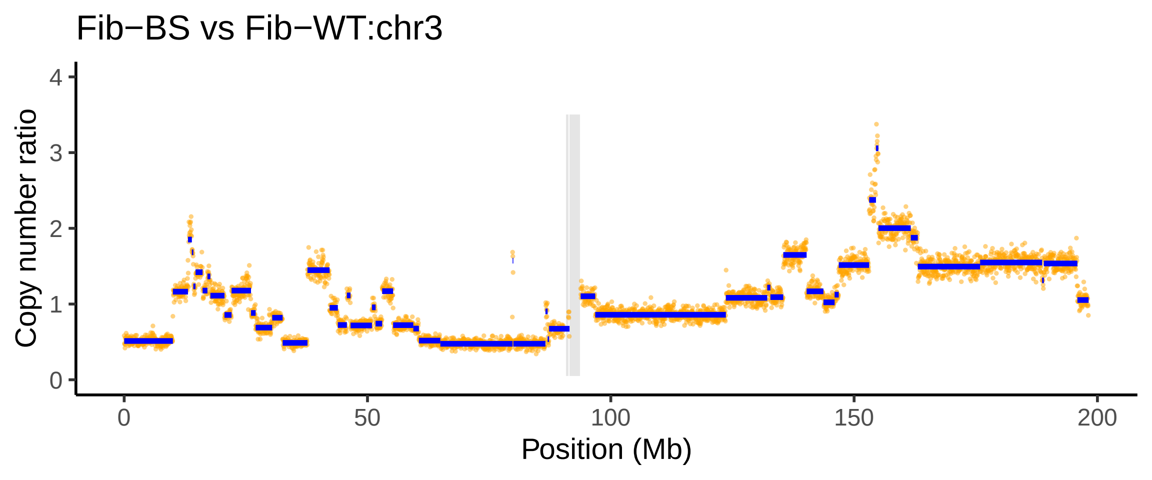
Fib−BS vs Fib−WT:chr3
Copy number ratio
0
1
2
3
4
0
50
100
150
200
Position (Mb)

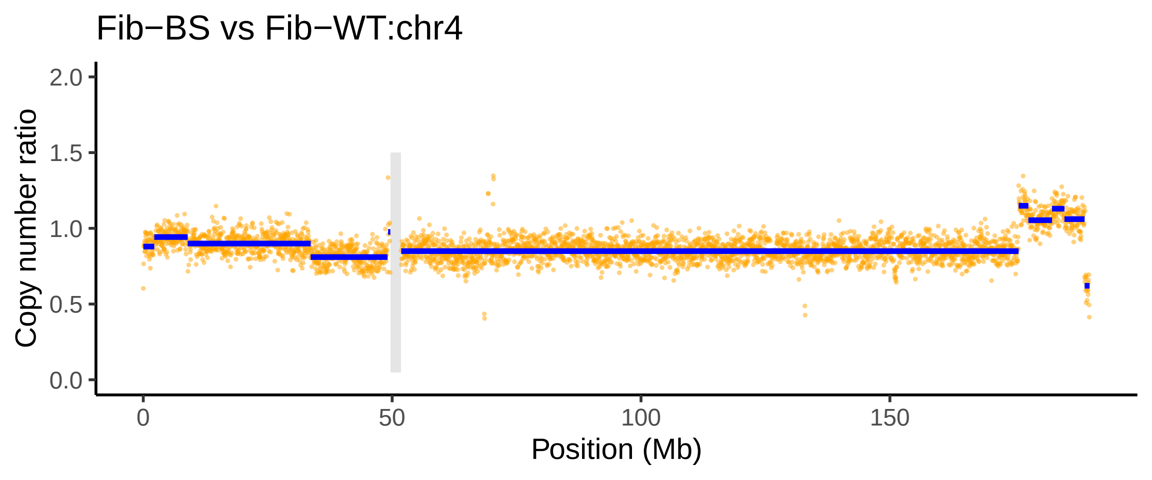
Fib−BS vs Fib−WT:chr4
Copy number ratio
2.0
1.5
1.0
0.5
0.0
0
50
100
150
Position (Mb)

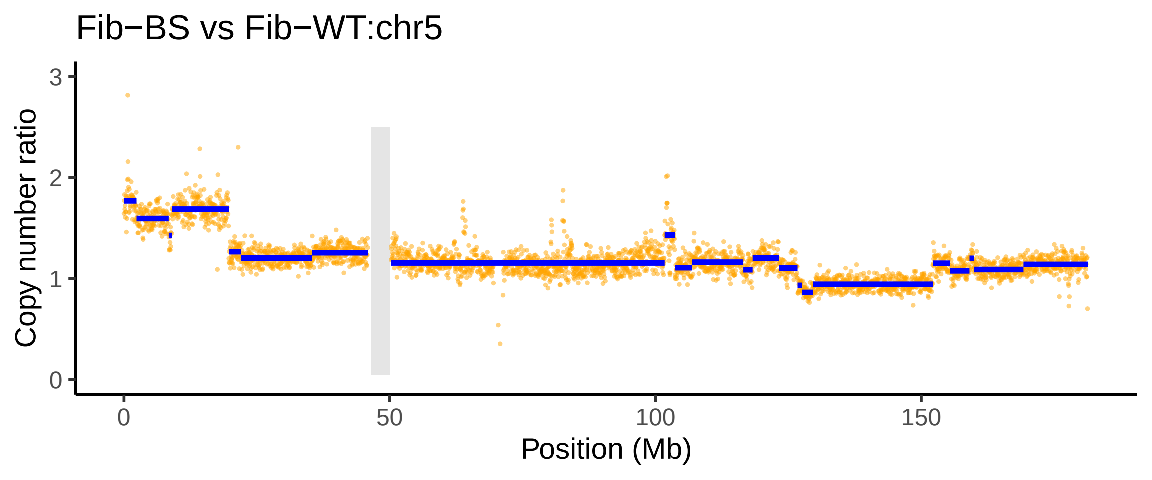
Fib−BS vs Fib−WT:chr5
Copy number ratio
0
1
2
3
0
50
100
150
Position (Mb)

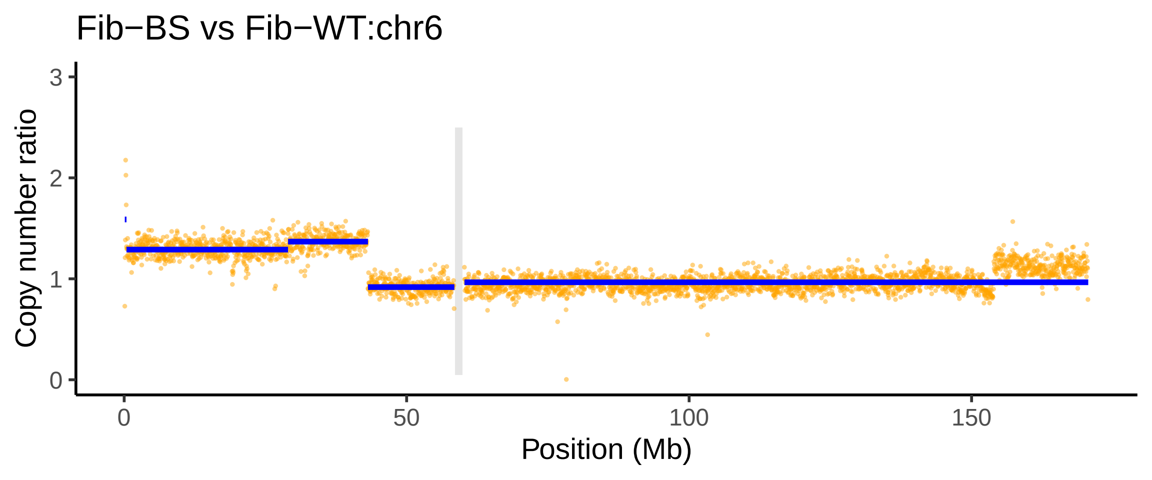
Fib−BS vs Fib−WT:chr6
Copy number ratio
0
1
2
3
0
50
100
150
Position (Mb)

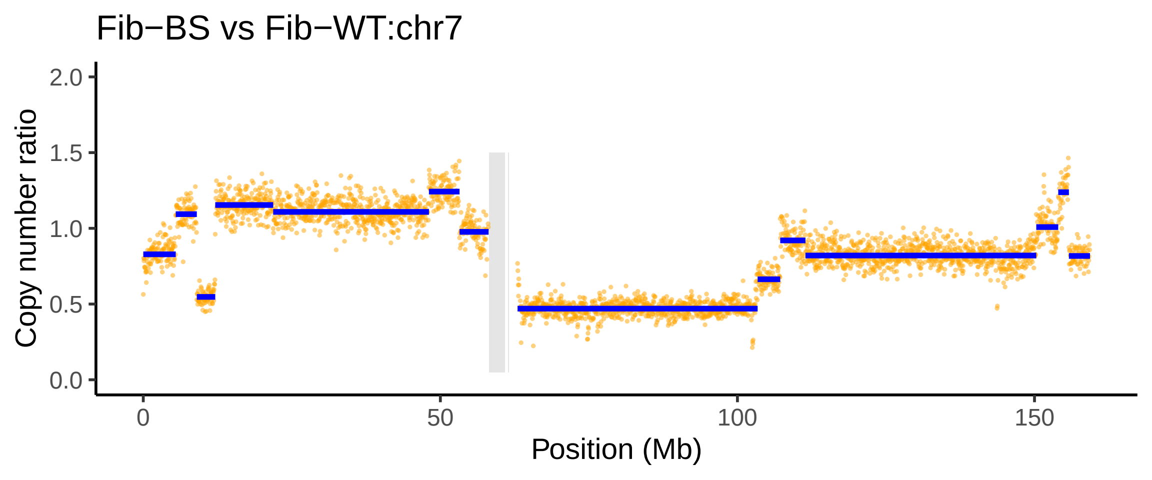

Fib−BS vs Fib−WT:chr7
Copy number ratio
2.0
1.5
1.0
0.5
0.0
0
50
100
150
Position (Mb)

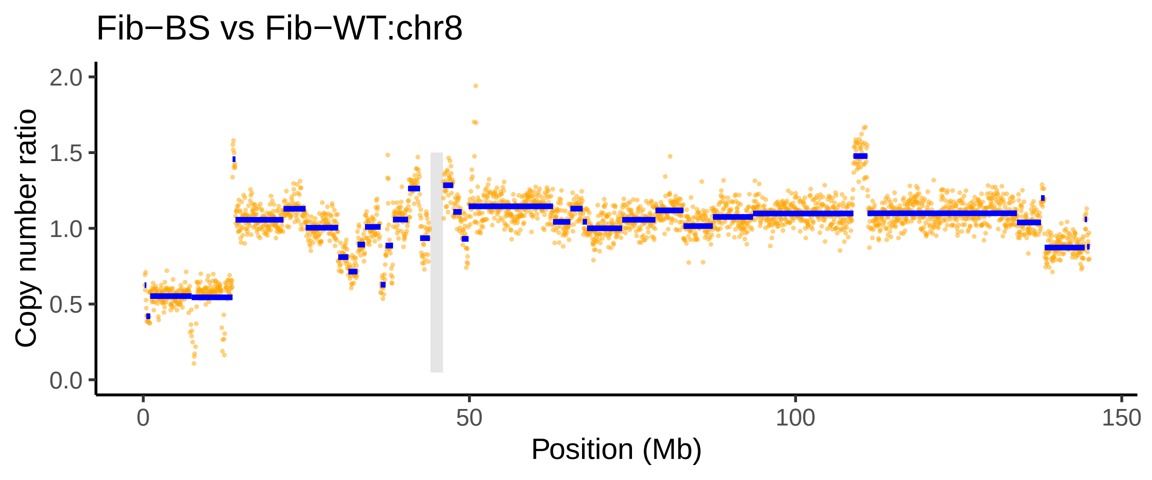

Fib−BS vs Fib−WT:chr8
Copy number ratio
2.0
1.5
1.0
0.5
0.0
0
50
100
150
Position (Mb)

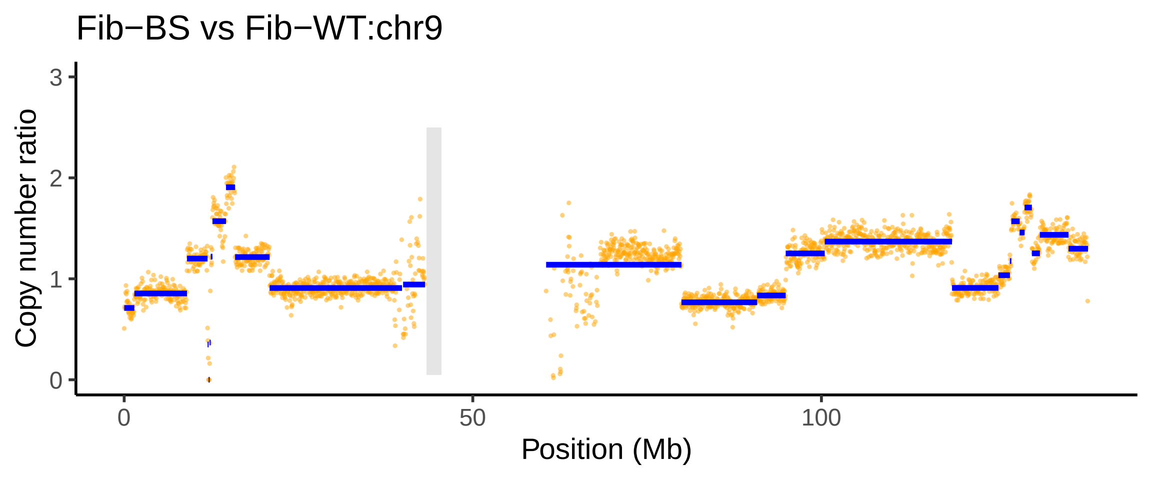

Fib−BS vs Fib−WT:chr9
Copy number ratio
0
1
2
3
0
50
100
Position (Mb)

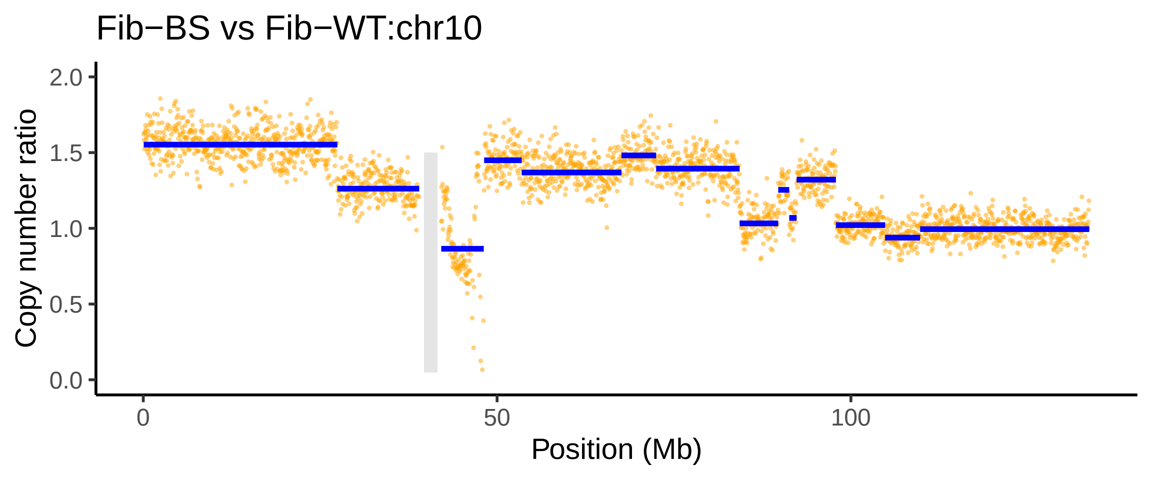

Fib−BS vs Fib−WT:chr10
Copy number ratio
2.0
1.5
1.0
0.5
0.0
0
50
100
Position (Mb)

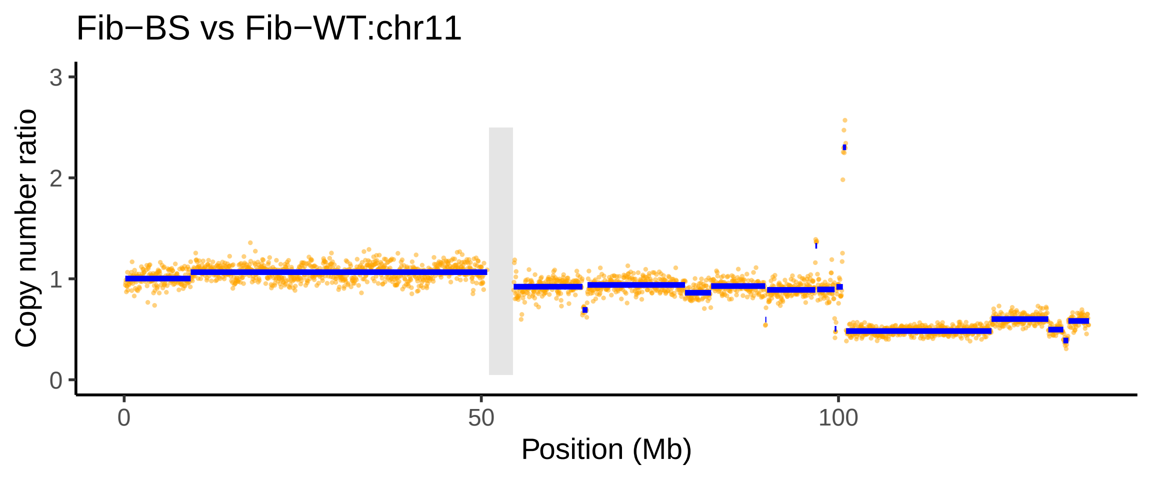
Fib−BS vs Fib−WT:chr11
Copy number ratio
0
1
2
3
0
50
100
Position (Mb)

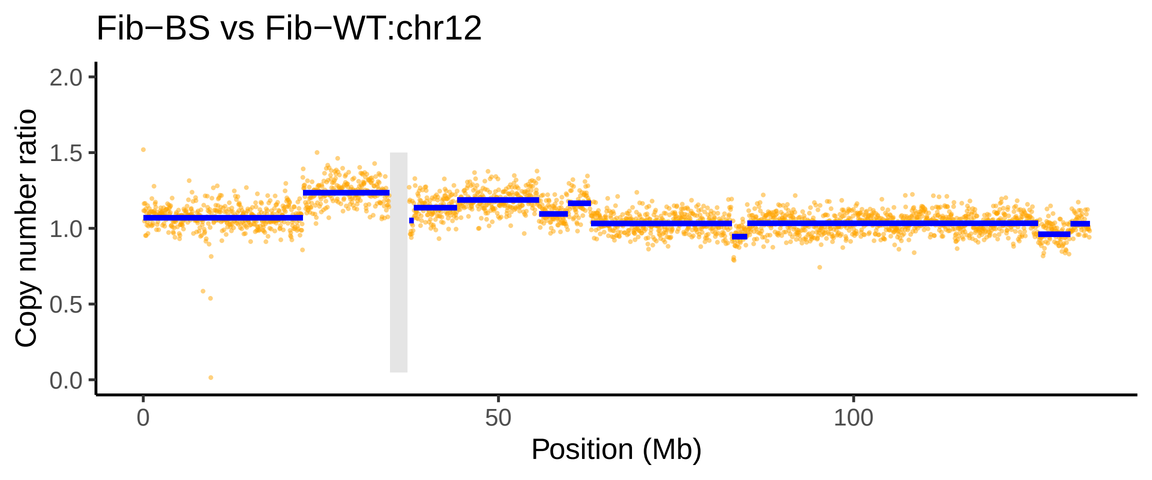

Fib−BS vs Fib−WT:chr12
Copy number ratio
2.0
1.5
1.0
0.5
0.0
0
50
100
Position (Mb)

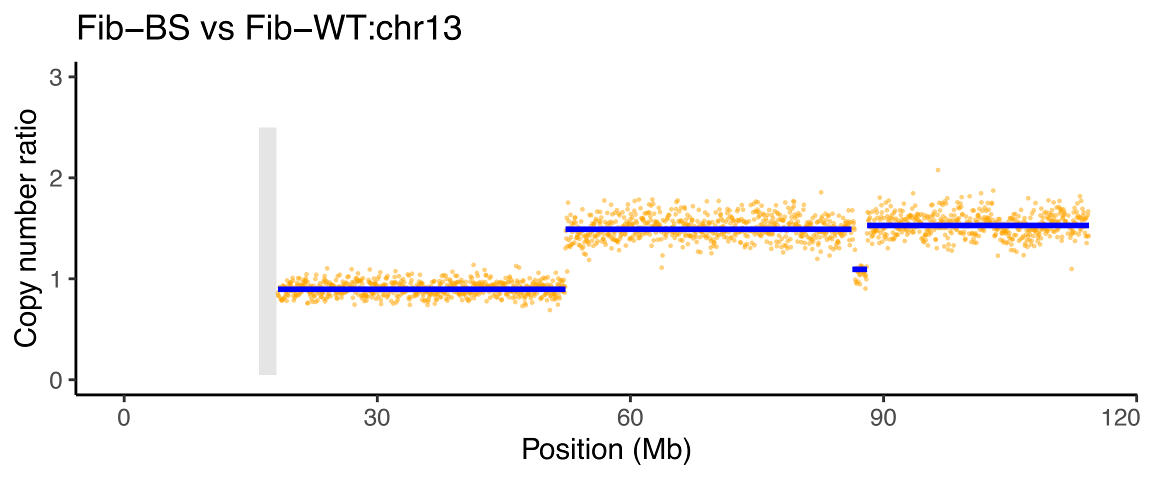
Fib−BS vs Fib−WT:chr13
Copy number ratio
0
1
2
3
0
30
60
90
120
Position (Mb)

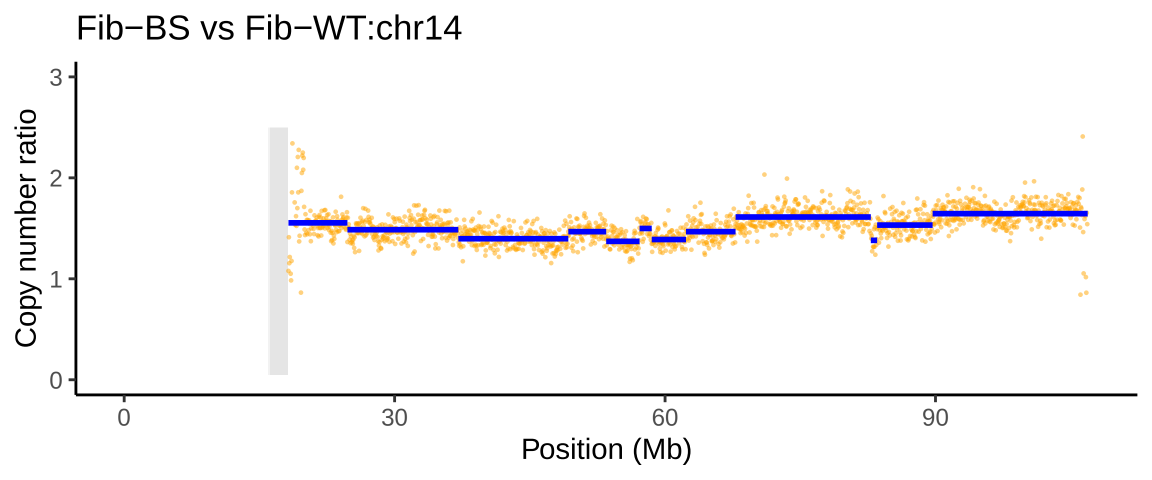
Fib−BS vs Fib−WT:chr14
Copy number ratio
0
1
2
3
0
30
60
90
Position (Mb)

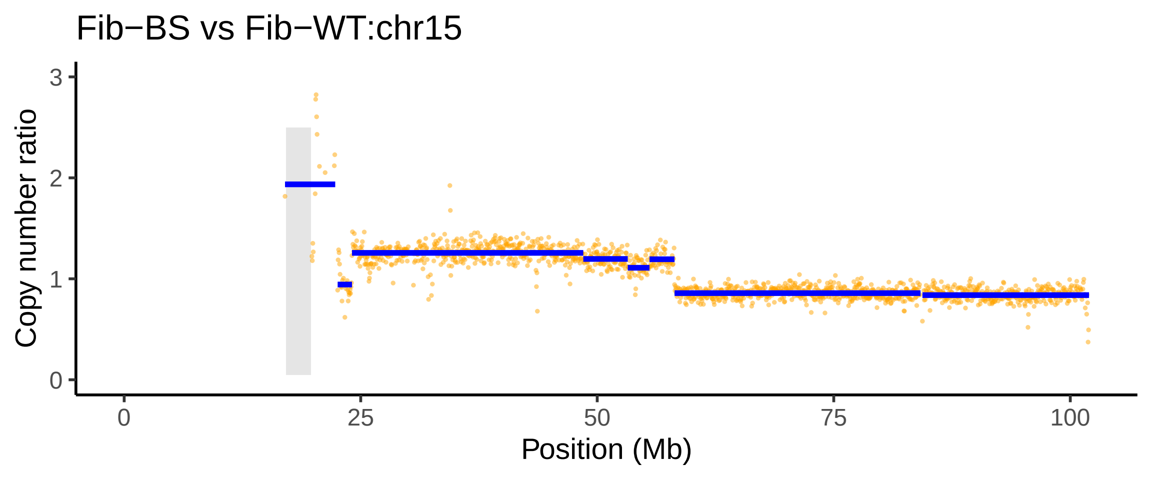
Fib−BS vs Fib−WT:chr15
Copy number ratio
0
1
2
3
0
25
50
75
100
Position (Mb)

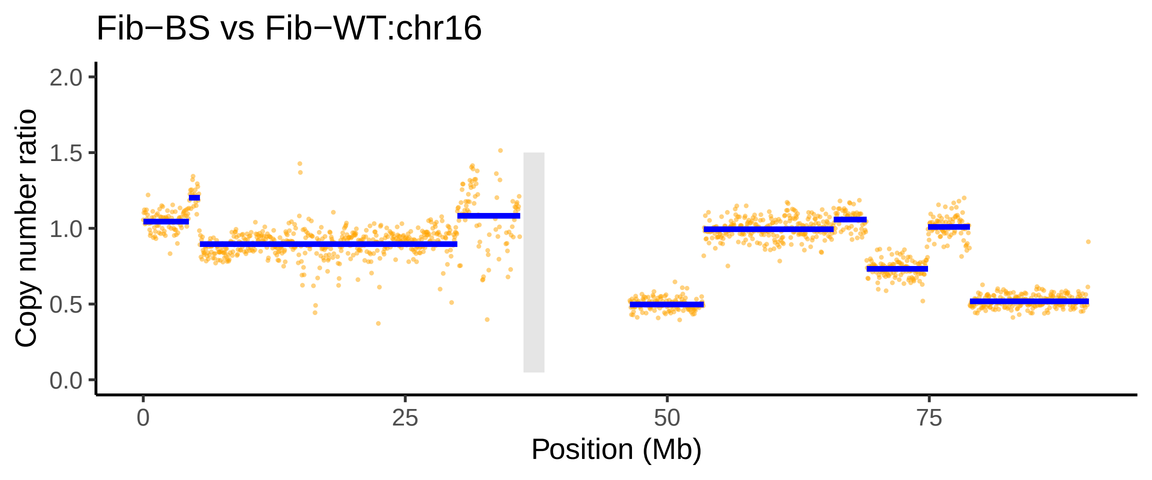
Fib−BS vs Fib−WT:chr16
Copy number ratio
2.0
1.5
1.0
0.5
0.0
0
25
50
75
Position (Mb)

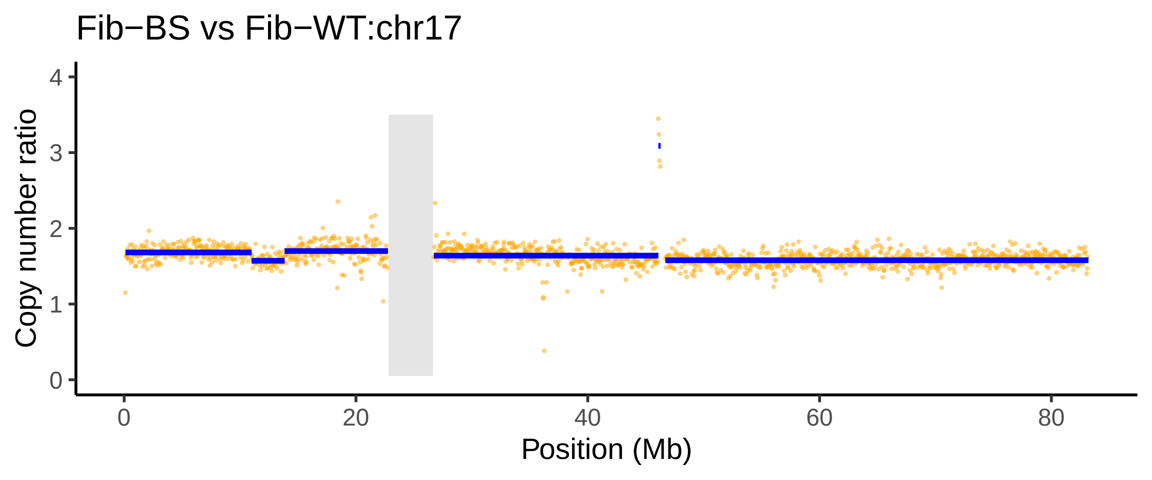

Fib−BS vs Fib−WT:chr17
Copy number ratio
0
1
2
3
4
0
20
40
60
80
Position (Mb)

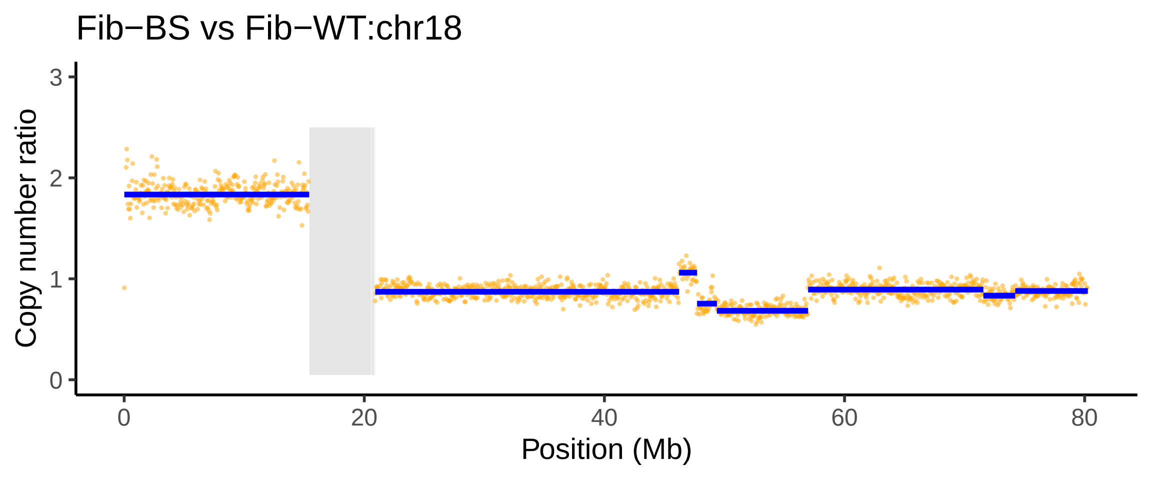

Fib−BS vs Fib−WT:chr18
Copy number ratio
0
1
2
3
0
20
40
60
80
Position (Mb)

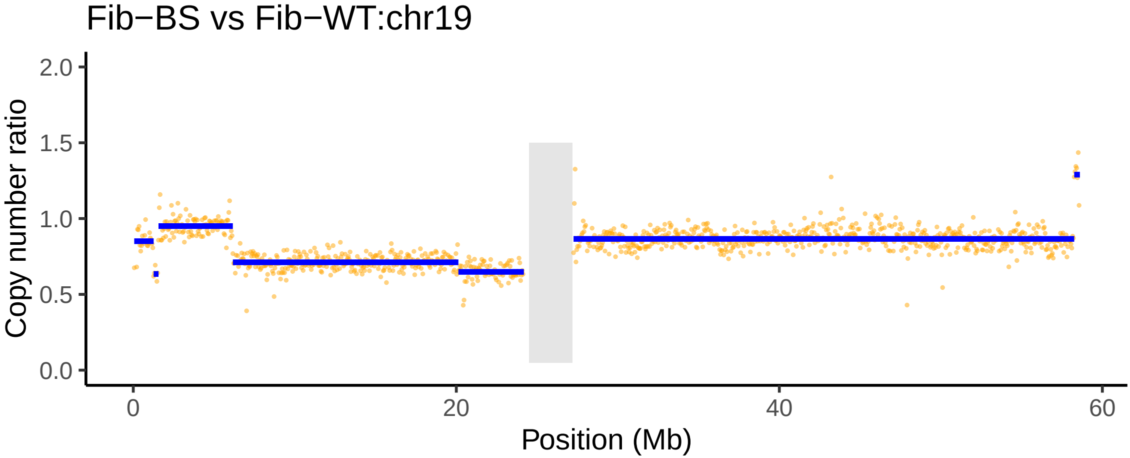
Fib−BS vs Fib−WT:chr19
Copy number ratio
2.0
1.5
1.0
0.5
0.0
0
20
40
60
Position (Mb)

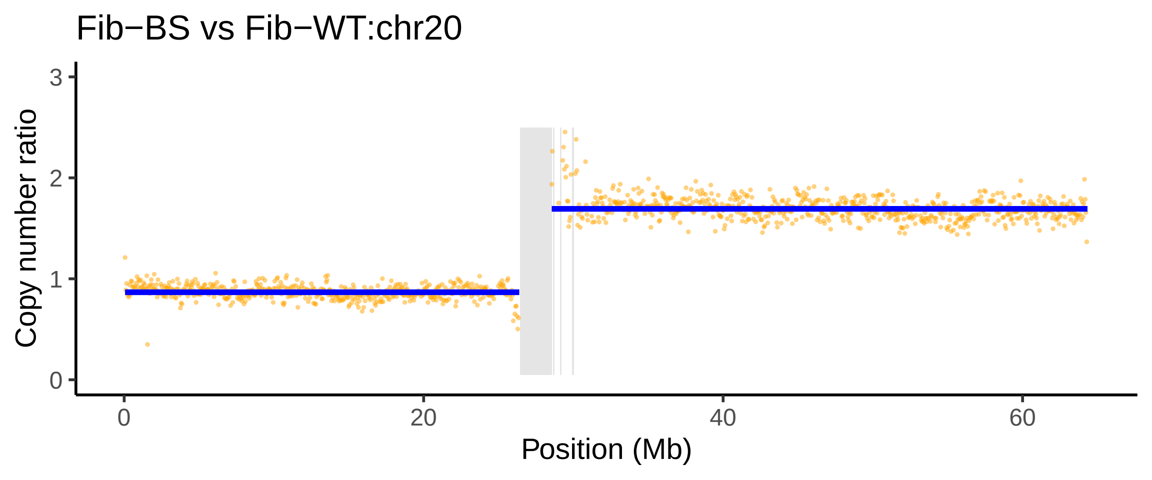
Fib−BS vs Fib−WT:chr20
Copy number ratio
0
1
2
3
0
20
40
60
Position (Mb)

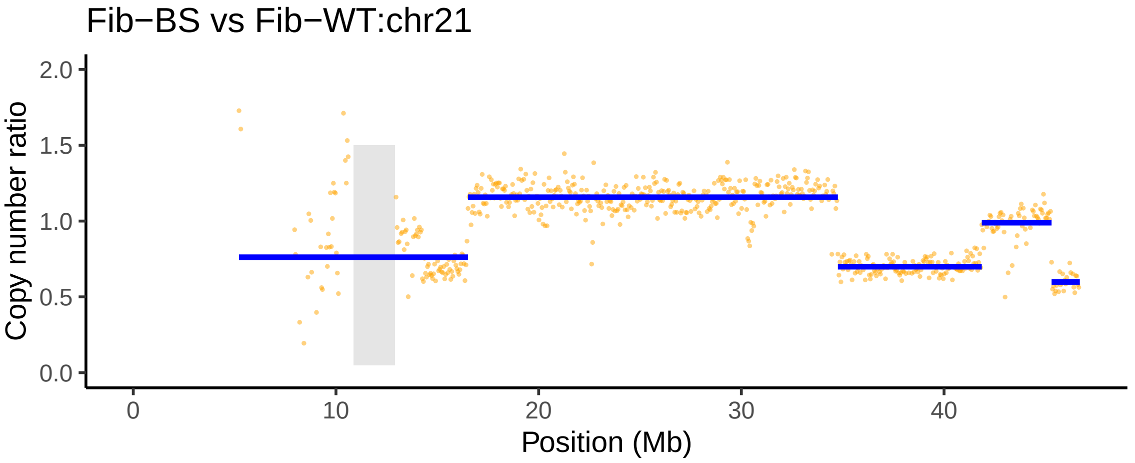

Fib−BS vs Fib−WT:chr21
Copy number ratio
0.0
0.5
1.0
1.5
2.0
0
10
20
30
40
Position (Mb)

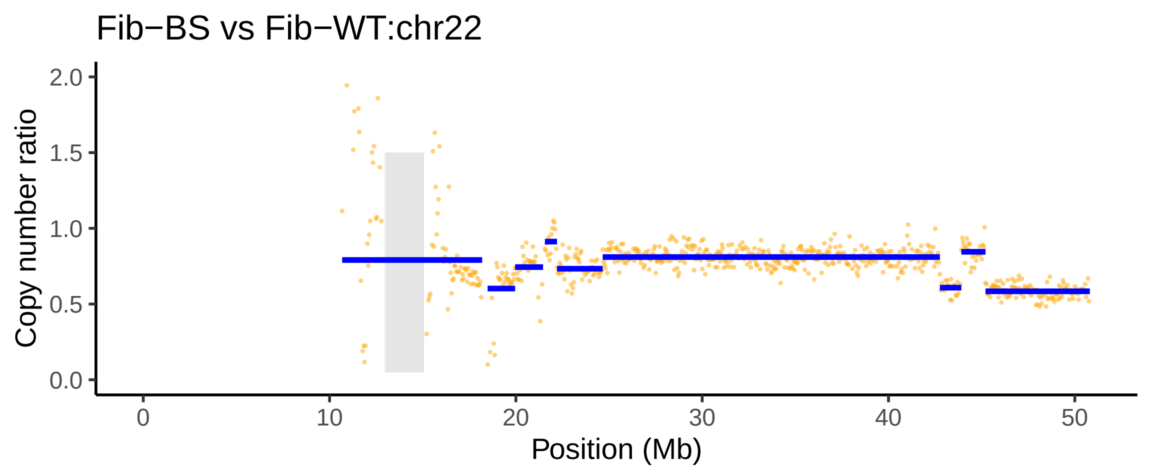
Fib−BS vs Fib−WT:chr22
Copy number ratio
2.0
1.5
1.0
0.5
0.0
0
10
20
30
40
50
Position (Mb)

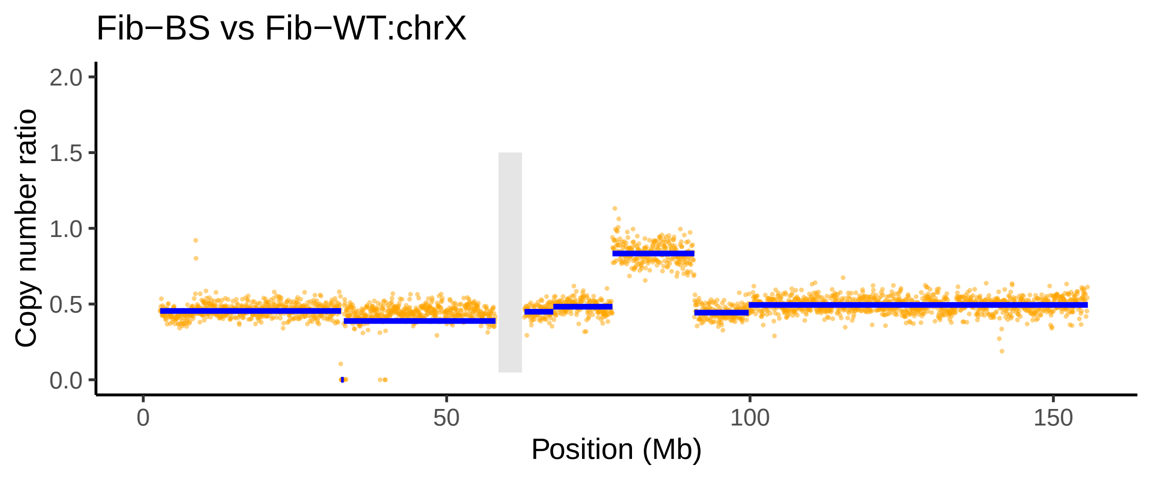

Fib−BS vs Fib−WT:chrX
Copy number ratio
2.0
1.5
1.0
0.5
0.0
0
50
100
150
Position (Mb)

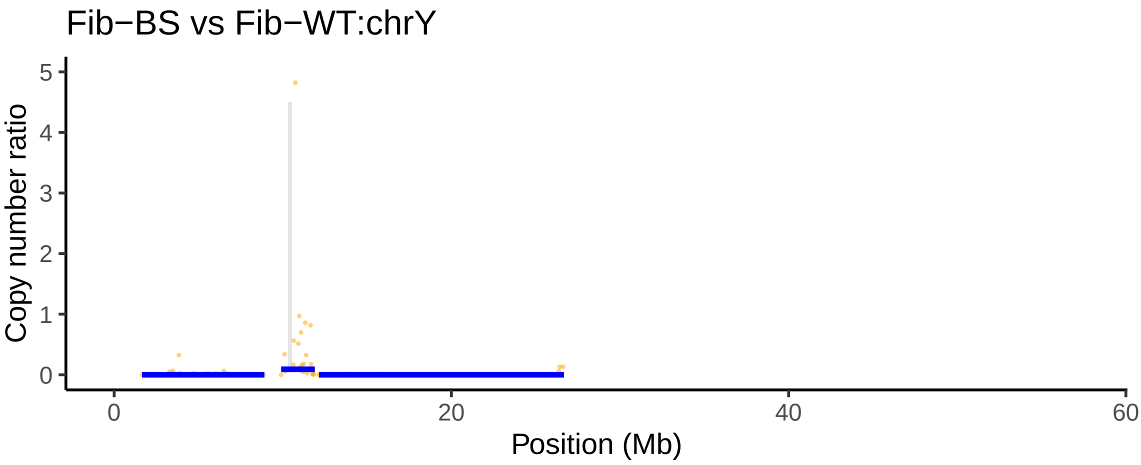

Fib−BS vs Fib−WT:chrY
Copy number ratio
0
1
2
3
4
5
0
20
40
60
Position (Mb)
