# Supplementary material for: Copy number normalization distinguishes differential signals driven by copy number differences in ATAC-seq and ChIP-seq
Source: BMC Genomics. 2025 Mar 28;26:306. doi: 10.1186/s12864-025-11442-y (PMC11951689; doi:10.1186/s12864-025-11442-y)

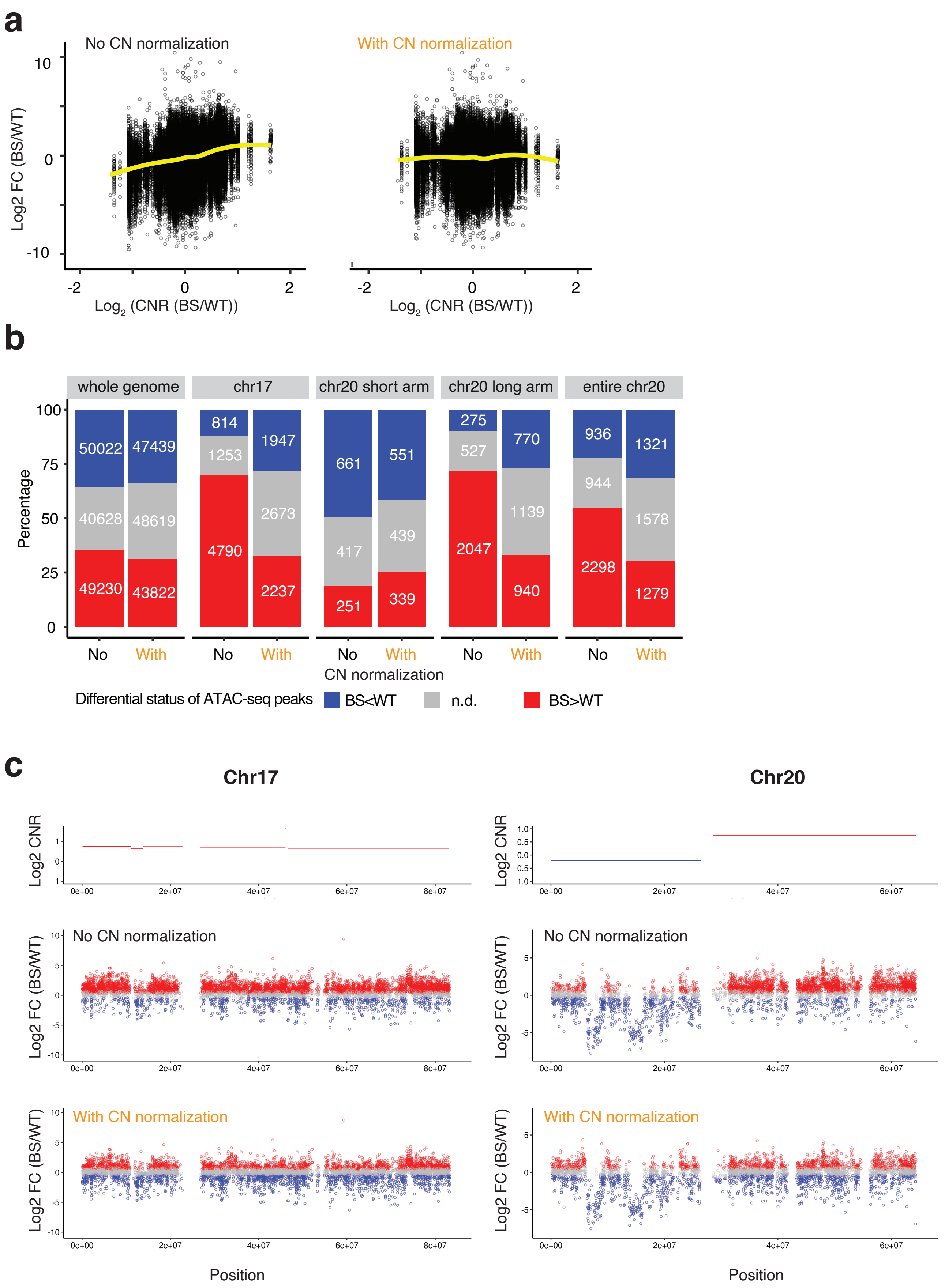

a
No CN normalization
With CN normalization
Log2 FC (BS/WT)
Log2 (CNR (BS/WT))
b
whole genome
chr17
chr20 short arm
chr20 long arm
entire chr20
Percentage
50022
47439
40628
48619
49230
43822
814
1253
4790
1947
2673
2237
661
417
251
551
439
339
275
527
2047
770
1139
940
936
944
2298
1321
1578
1279
No
With
CN normalization
Differential status of ATAC-seq peaks
BS<WT
n.d.
BS>WT
c
Chr17
Chr20
Log2 CNR
Log2 FC (BS/WT)
No CN normalization
With CN normalization
Position

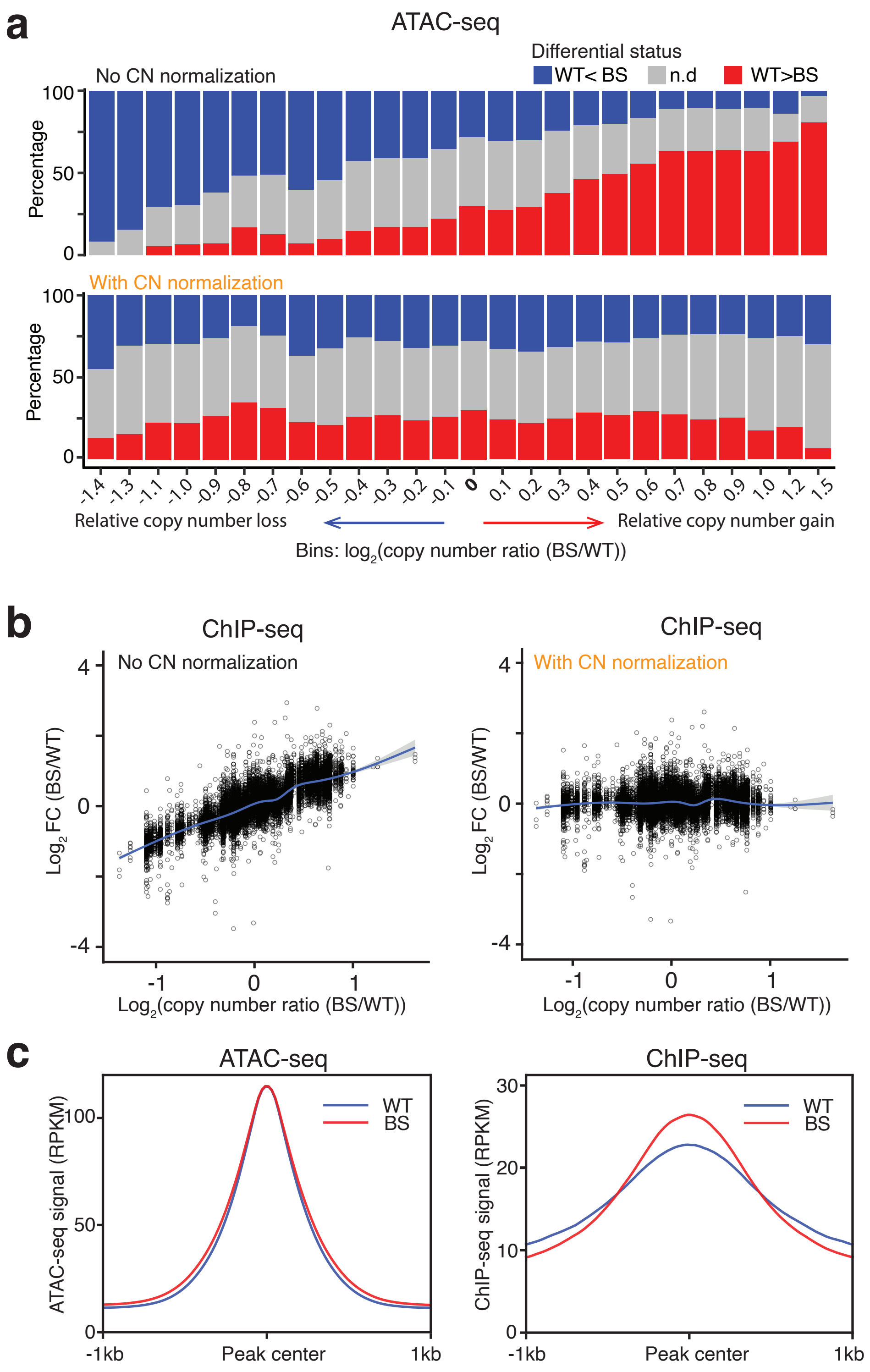

a
ATAC-seq
Differential status
WT< BS
n.d
WT>BS
No CN normalization
Percentage
100
50
0
With CN normalization
-1.4 -1.3 -1.1 -1.0 -0.9 -0.8 -0.7 -0.6 -0.5 -0.4 -0.3 -0.2 -0.1 0 0.1 0.2 0.3 0.4 0.5 0.6 0.7 0.8 0.9 1.0 1.2 1.5
Relative copy number loss
Relative copy number gain
Bins: log2(copy number ratio (BS/WT))
b
ChIP-seq
No CN normalization
With CN normalization
Log2 FC (BS/WT)
4
0
-4
-1
1
Log2(copy number ratio (BS/WT))
c
ATAC-seq
ChIP-seq
WT
BS
ATAC-seq signal (RPKM)
100
50
ChIP-seq signal (RPKM)
30
20
10
-1kb
Peak center
1kb

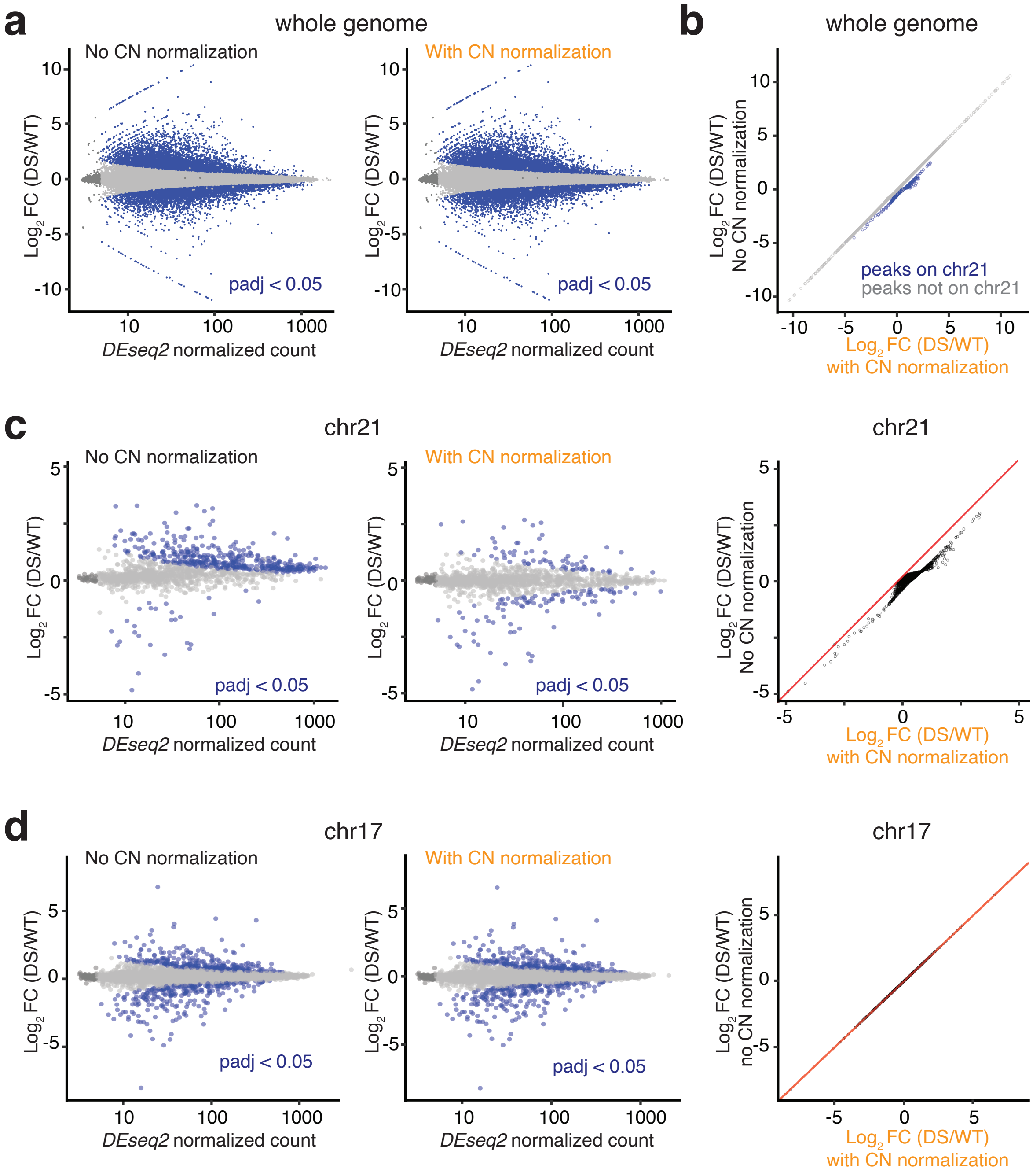

a
whole genome
No CN normalization
With CN normalization
Log2 FC (DS/WT)
padj < 0.05
DEseq2 normalized count
10
100
1000
b
whole genome
Log2 FC (DS/WT) No CN normalization
peaks on chr21
peaks not on chr21
Log2 FC (DS/WT) with CN normalization
c
chr21
No CN normalization
With CN normalization
Log2 FC (DS/WT)
padj < 0.05
DEseq2 normalized count
chr21
Log2 FC (DS/WT) No CN normalization
Log2 FC (DS/WT) with CN normalization
d
chr17
No CN normalization
With CN normalization
Log2 FC (DS/WT)
padj < 0.05
DEseq2 normalized count
chr17
Log2 FC (DS/WT) no CN normalization
Log2 FC (DS/WT) with CN normalization

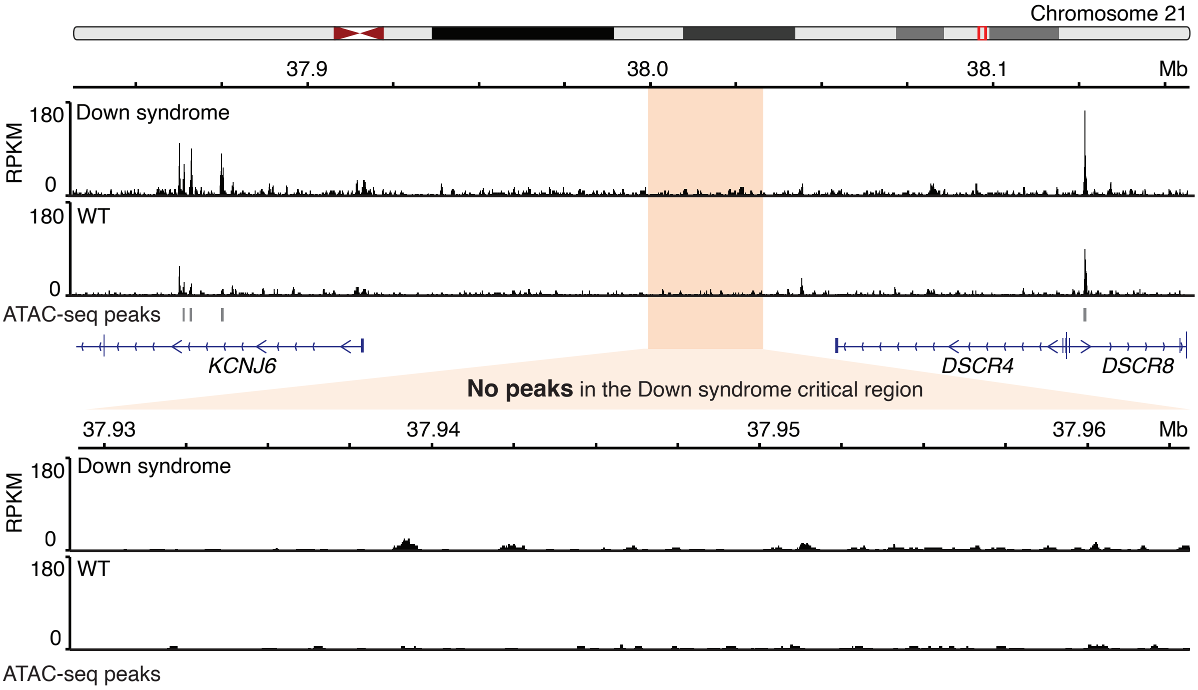
Chromosome 21
37.9
38.0
38.1
Mb
RPKM
180
0
Down syndrome
180
0
WT
ATAC-seq peaks
KCNJ6
DSCR4
DSCR8
No peaks in the Down syndrome critical region
37.93
37.94
37.95
37.96
Mb
RPKM
180
0
Down syndrome
180
0
WT
ATAC-seq peaks

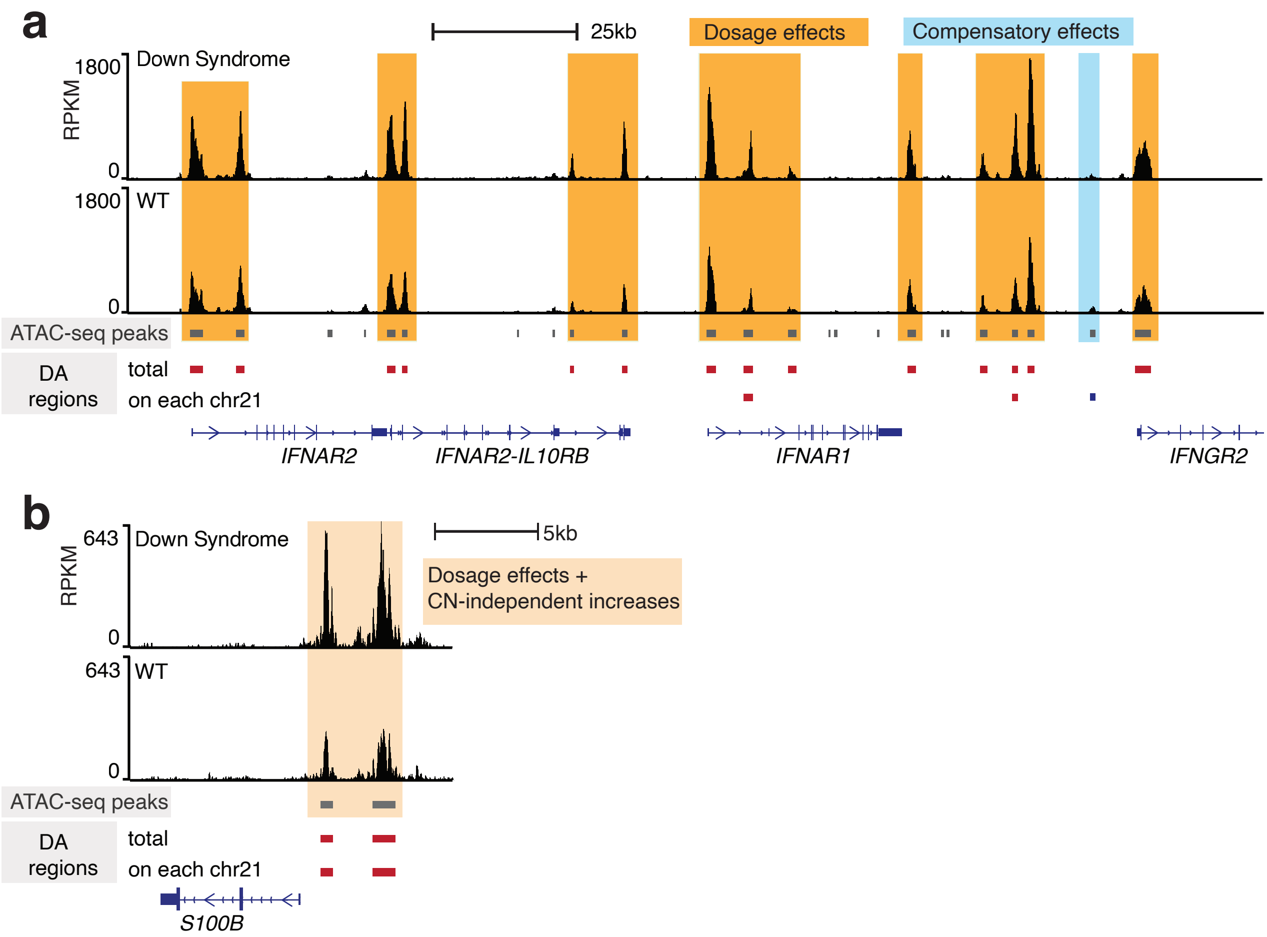
a
25kb
Dosage effects
Compensatory effects
RPKM
1800
0
Down Syndrome
1800
0
WT
ATAC-seq peaks
DA regions
total
on each chr21
IFNAR2
IFNAR2-IL10RB
IFNAR1
IFNGR2
b
RPKM
643
0
Down Syndrome
643
0
WT
5kb
Dosage effects +
CN-independent increases
ATAC-seq peaks
DA regions
total
on each chr21
S100B

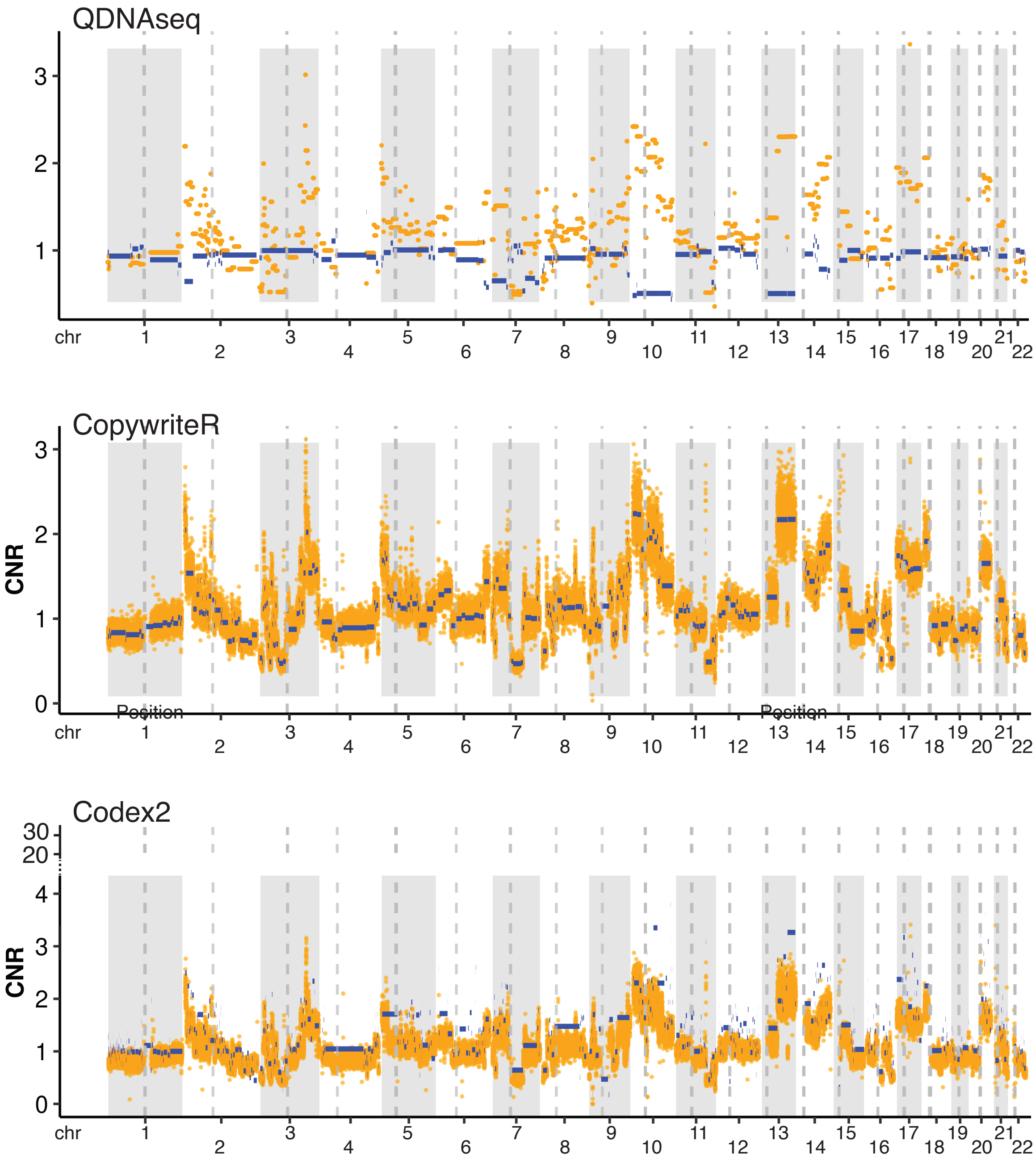
QDNAseq
CopywriteR
Codex2
CNR
Position
chr
1 2 3 4 5 6 7 8 9 10 11 12 13 14 15 16 17 18 19 20 21 22

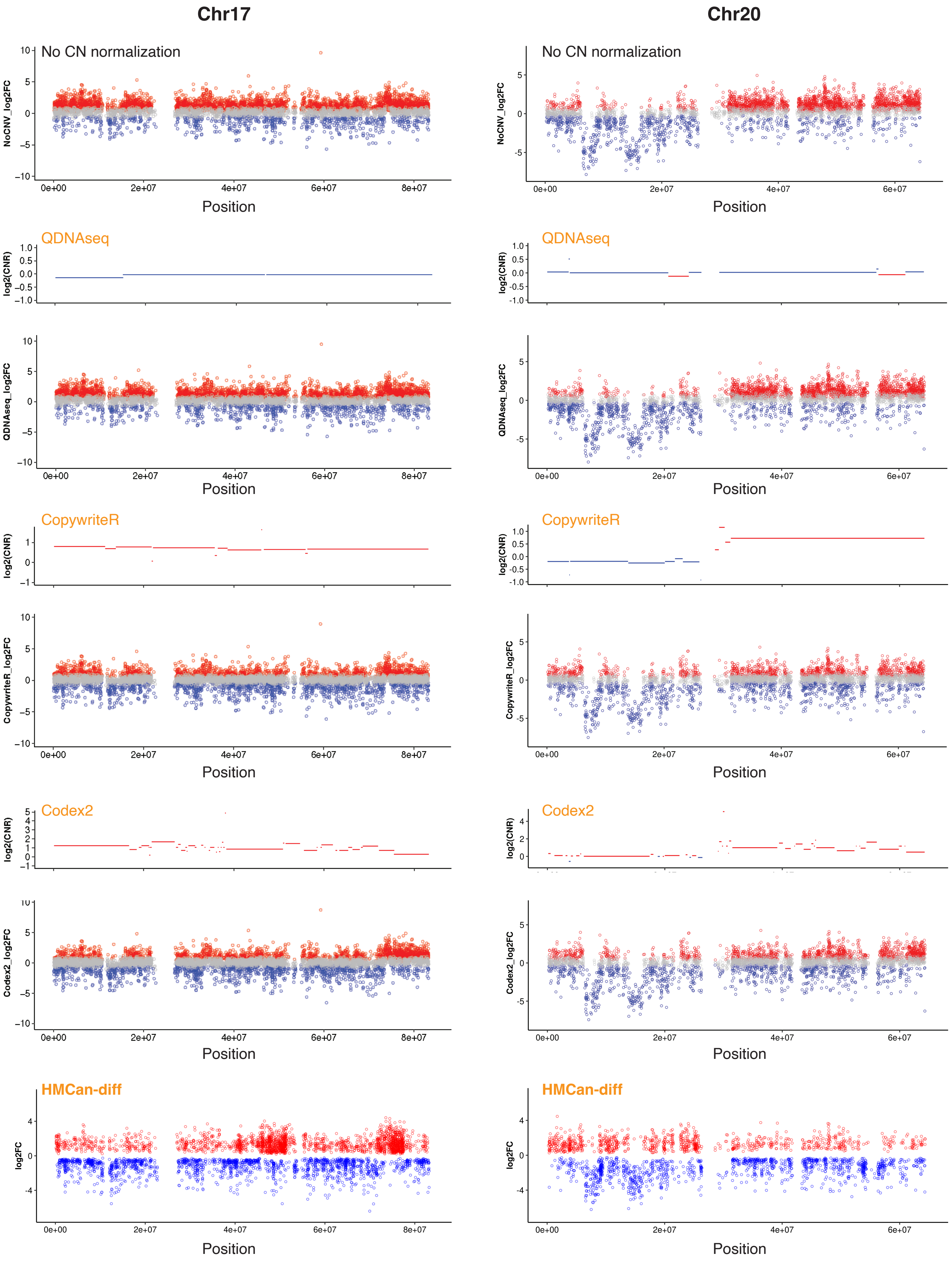
Chr17
Chr20
No CN normalization
NoCNV_log2FC
Position
QDNAseq
log2(CNR)
QDNAseq_log2FC
CopywriteR
CopywriteR_log2FC
Codex2
Codex2_log2FC
HMCan-diff
log2FC
0e+00
2e+07
4e+07
6e+07
8e+07

a

Example of HMCan-diff "peaks" and "regions" files:

Chromosome	Start	End	Name	Score	Strand	Differential state	log2(density fold change)
chr1	219297349	219301500	peak16239	54.009502	.	condition2	-1.762861
chr1	219406199	219410700	peak16246	56.637878	.	condition2	-1.665246
chr1	219515549	219520000	peak16251	55.166691	.	condition2	-2.050705
chr1	235478399	235484650	peak16886	42.370319	.	condition1	1.425381
chr1	235591299	235597300	peak16888	39.039383	.	condition1	1.344224
chr1	235704249	235710450	peak16896	70.569687	.	condition1	1.411959

b

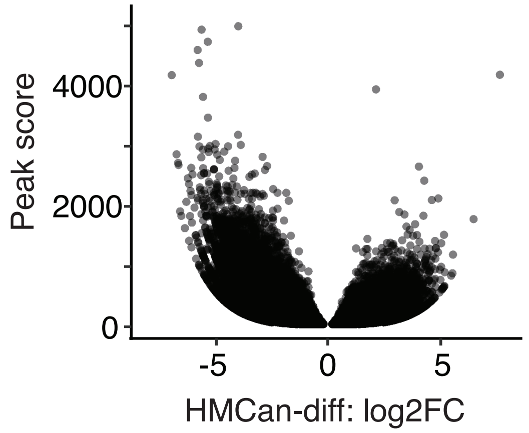

Supplement: Supplementary file 2 — Additional file 2: Supplementary Fig. 1. Copy number variation drives the differential signals in ATAC-seq data upon using edgeR. (a) Trends in differential chromatin accessibility biases caused by copy number differences, shown without (left) and with (right) copy number (CN) normalization. Yellow lines represent the generalized additive model (GAM) fitted to the data, with the grey shaded area indicating the 95% confidence interval for the fitted curve. Log2CNR > 0 and log2CNR< 0 indicate regions with relative number gain and loss in BS, respectively. (b) The number and proportion of differential ATAC-seq peaks across the genome, on chr17, and on chr20, before and after applying CN normalization. (c) Averaged signal profiles of ATAC-seq and ChIP-seq in their corresponding peak sets. Supplementary Fig. 2. Impacts of copy number normalization on the differential analysis of ATAC-seq and ChIP-seq. (a) The proportion of more accessible, non-differential and less accessible ATAC-seq peaks in regions with different CNR without (top) and with (bottom) applying copy number (CN) normalization. Log2CNR> 0 and log2CNR < 0 indicate regions with relative number gain and loss in BS, respectively. (b) The trended biases of detected differential G-quadruplex formation activities driven by copy number differences without (left) and with (right) applying copy number normalization in ChIP-seq data. (c) Averaged signal profiles of ATAC-seq and ChIP-seq in their corresponding peak sets. Supplementary Fig. 3. Impacts of copy number normalization on differential anlaysis of Down syndrome ATAC-seq data. (a) MA plots of the genome-wide differential signals without (left) and with (right) applying copy number (CN) normalization. Differential peaks with adjusted P (p-adj) < 0.05 are depicted in blue while those with p-adj ≥ 0.05 are shown in grey. (b) A comparison of the differential signals without and with applying CN normalization for peaks on chromosome 21 (chr21) (blue) and other chrom [file 12864_2025_11442_MOESM2_ESM.pdf]
