# Supplementary material for: Copy number normalization distinguishes differential signals driven by copy number differences in ATAC-seq and ChIP-seq
Source: BMC Genomics. 2025 Mar 28;26:306. doi: 10.1186/s12864-025-11442-y (PMC11951689; doi:10.1186/s12864-025-11442-y)
Supplement: Supplementary file 4 — Additional file 4: Supplementary Table 1. Copy number awareness of commonly used tools in ATAC-seq and ChIP-seq differential analyses [77–81]. Notes: *background signal awareness when the genomic sequencing or ChIP-seq input data is available; n/a, not applicable. [file 12864_2025_11442_MOESM4_ESM.docx]

| **Tools** | **Purpose** | **Background signal awareness (*)** | **Copy number awareness** | | **Comments** | **Ref.** |
| --- | --- | --- | --- | --- | --- | --- |
|  |  |  | **Within sample** | **Across**  **sample** |  |  |
| *MACS2, MACS3* | Peak calling | Yes | No | n/a | Intended for ChIP-seq;  A popular peak caller for ATAC-seq and ChIP-seq data. | [10] |
| *JAMM* |  | Yes | No | n/a | Intended for ChIP-seq. | [77] |
| *SICER* |  | Yes | No | n/a | Intended for ChIP-seq. | [78] |
| *F-seq* |  | Yes | Yes | n/a | Intended for DNase-seq and ChIP-seq;  Available background models in the tool represent how many places a given sequence occurs in reference genomes;  Customized background models for individual samples correct for copy number/karyotype. | [79] |
| *HMMRATAC* |  | Yes | No | n/a | Intended for ATAC-seq;  Features of ATAC-seq signals from nucleosome-free and nucleosomal regions are learned in a Hidden Markov Model and subsequently used to predict genome-wide peaks. | [80] |
| *Genrich* |  | Yes | No | n/a | Intended for ATAC-seq. | [81] |
| *htseq-count* | Signal quantification | No | No | No | Requires an external peak set;  Counts background signals as enriched signals. | [15] |
| *featureCount* | Signal quantification  Data normalization and differential signal detection | No  Optional | No | No | Requires an external peak set;  Counts background signals as enriched signals.  Requires an external peak set;  Background signals can be sustracted depending on parameter choices. | [16] |
| *deepTools* |  |  | No | No |  | [14] |
| *bedtools* |  |  | No | No |  | [13] |
| *DiffBind* |  |  | No | No |  | [20] |
| *Csaw* |  | No | No | No | Does not requiring an external peakset;  Using a sliding windows approach. | [19] |
| *DESeq2* |  | No | No | No | A commonly used tool for the differential analyses of ATCA-seq, RNA-seq and ChIP-seq. | [18] |
| *edgeR* | Data normalization and differential signal detection  Data normalization | No | No | No | A commonly used tool for the differential analyses of ATCA-seq, RNA-seq and ChIP-seq. | [17] |
| *loess*  (in *csaw*) |  | No | No | No | Usually followed by dfifferential signal detection using the methods integrated in *DESeq2* or *edgeR.* | [19] |
| *MACS2-bgddiffm* | Differential peak calling with external peaksets | Yes | No | No | Background signals are substracted from the enriched signals. | [10] |

**Supplementary Table 1**. Copy number awareness of commonly used tools in ATAC-seq and ChIP-seq differential analyses.

Notes: *, background signal awareness when the genomic sequencing or ChIP-seq input data is available; n/a, not applicable.
